# Attention's grasp: early and late hand proximity effects on visual evoked potentials

**DOI:** 10.3389/fpsyg.2013.00420

**Published:** 2013-07-12

**Authors:** Catherine L. Reed, David S. Leland, Benjamin Brekke, Alan A. Hartley

**Affiliations:** ^1^Cognitive Neuroscience Laboratory, Department of Psychology, Claremont McKenna CollegeClaremont, CA, USA; ^2^Department of Psychology, University of Wisconsin - Eau ClaireEau Claire, WI, USA; ^3^Department of Psychology, Pitzer CollegeClaremont, CA, USA; ^4^Adult Development Laboratory, Department of Psychology, Scripps CollegeClaremont, CA, USA

**Keywords:** attention, multisensory, bimodal, visuo-tactile, event-related potentials (ERPs)

## Abstract

Behavioral studies suggest that visual attention is biased toward stimuli in the region of space near the palm of the hand, but it is unclear whether this effect is universal or selective for goal/task-related stimuli. We examined event-related potentials (ERPs) using a visual detection task in which the hand was placed near or kept far from target and non-target stimuli that were matched for frequency and visual features to avoid confounding factors. Focusing on attention-sensitive ERP components, we found that P3 (350–450 ms) amplitudes were increased for Hand Near conditions for targets only, demonstrating a selective effect consistent with the P3's cross-modal and task-relevance influences. An N1 variant implicated in visuo-tactile integration (central Nd1; 120–190 ms) showed similar target-specific effects. P1 (80–110 ms) effects for target stimuli were also apparent, but may have applied to non-targets as well, which would be consistent with the P1's association with early, pre-categorical increases in sensory gain. Collectively, these findings suggest that by the time stimuli are categorized as relevant/irrelevant for action, the proprioceptive effects of the hand on visual attention are selective for goal/task-related stimuli. At the same time, hand proximity appears to bias attention early, starting with a facilitation of processing for perhaps any visual stimuli near the hand, and continuing with enhancements that are selective to those stimuli categorized as task-relevant.

The embodied view in cognitive science focuses on how the functional capabilities of the body influence information-processing (Wilson, 2001). For example, the hands may have an impact on attention because they are the main effectors by which we interact with the world. The region of space near the palm represents a more likely candidate for action than other locations, and as such may command a disproportionate share of attentional resources (Reed et al., 2007). Behavioral studies have demonstrated such attentional biases (Reed et al., 2006, 2010; Abrams et al., 2008; Cosman and Vecera, 2010; Tseng and Bridgeman, 2011), but it is unclear at what stage of cognitive processing hand proximity becomes influential for spatial attention and how it affects processing. These questions are well-suited to an electroencephalography (EEG) approach, which has the temporal resolution necessary to reveal when stimuli near the hand receive increased neural resources. In this study, we examined event-related potentials (ERPs) to target and non-target stimuli presented near and far from the hand to determine whether having stimuli in grasping space facilitates attention for all stimuli in the same manner or whether some effects are selective to goal/task-relevant stimuli.

In two covert orienting studies, Reed and colleagues demonstrated a facilitation of processing for targets in grasping space, even when hand position was unrelated to task demands (Reed et al., 2006, 2010). For example, in covert orienting tasks, participants detected the onset of targets at peripheral locations of a computer screen more rapidly when the hand was held up near that location than when the hand was held away. This effect occurred even when the hand was visually occluded, but was eliminated when an arbitrary visual anchor (a board) was placed next to potential target locations instead of the hand (Reed et al., 2006). Thus, these results suggest that it is not the visual stimulus of the hand, but rather tactile/proprioceptive information about hand position that affects the processing of visual targets in grasping space. Nonetheless, behavioral studies cannot definitively determine at what stage of processing such visual and tactile/proprioceptive sensory integration occurs.

Some behavioral studies suggest that the hand's influence occurs early in processing (Abrams et al., 2008; Cosman and Vecera, 2010). Hands positioned close to the location of a visual stimulus can slow the shifting of attention away from that location in a variety of attention tasks, such as covert attention, inhibition of return, and attentional blink tasks (Abrams et al., 2008), and can improve visual short-term working memory (Tseng and Bridgeman, 2011). Hand proximity also affects figure-ground segregation, a process thought to occur early in visual processing (Cosman and Vecera, 2010). When participants placed either their hand or a wooden dowel on one region of two-color ambiguous figure, the region near the hand, but not the dowel, was more likely to be perceived as an object than background. Such studies demonstrate the integration of visual inputs with tactile and proprioceptive inputs for stimuli appearing in grasping space. In these studies, the conditions under which hand bias is found are consistent with those recording from visuo-tactile bimodal neurons in non-human primate single-cell recording studies: These neurons respond to tactile stimuli on the hand as well as visual stimuli presented on or near the hand (Graziano and Gross, 1993; Graziano, 1999; Graziano and Cooke, 2006) and are located in cortical regions that support a multimodal system for upcoming action: parietal cortex, premotor cortex, and the putamen (Graziano and Gross, 1994; Fogassi et al., 1996; Duhamel et al., 1998; Graziano, 1999; Graziano and Cooke, 2006). These sensory effects are thought to occur early in sensory processing at pre-categorical levels.

Other studies demonstrate that hand position effects involve later, higher-order processing. For example, Davoli et al. (2010, 2012) have shown that hand-proximity can bias observers toward detail-oriented processing of nearby stimuli, although at the expense of general semantic processing. Garza et al. (2013) found a biasing effect of the hand on target detection when instructions emphasized the location of the hand held near targets but not when instructions emphasized the location of the other hand, which was used to make responses. Qian et al. (2012) found a biasing effect of the hand only when stimuli near it were task relevant. These studies suggest that hand presence itself may imply a task context, biasing participants' expectations as to where important stimuli may occur, but that top-down influences from instructions and task demands can likewise shape this context, ultimately improving the potential for functional interaction with objects.

Event-related potentials can reveal the time course and selectivity of hand position influences on visuospatial attention because we can look for an influence of the hand on ERP components known to reflect specific stages of cognitive processing. In this study, we used a target detection task designed to evoke a P3 as well as earlier components whose amplitudes could vary as a function of hand position. Thus, we focused on three attention-sensitive ERP components that have been implicated in both sensory and task-related aspects of visuo-tactile tasks: the P1, Nd1, and P3. The P1 (80–110 ms) is a positive deflection over lateral posterior regions of the scalp, reflecting the activity of extrastriate cortex generators (Hillyard et al., 1998). It has greater amplitude in response to stimuli at attended than unattended locations, and is thought to reflect early sensory gain control mechanisms. The Nd1 (150–200 ms) is a negative deflection and a variant of the N1, with a midline parietal distribution that has been implicated in visuo-tactile integration (Kennett et al., 2001). For instance, the Nd1 shows greater amplitudes in a cross-modal cuing task when visual and tactile stimuli were presented than when unimodal stimuli were presented. Likewise, a similar component was found to be enhanced when visual stimuli were presented on the hand for implicit “touch” as opposed to near the hand (Simon-Dack et al., 2009). Finally, the P3 (300–500 ms) reflects discrimination of stimulus categories at a more abstract, task or motivationally relevant level. It is typically maximal over centroparietal regions and is produced by a number of neural generators and cognitive factors including allocation of attentional resources and categorization of events (Kok, 2001). The P3 response to stimuli can vary by category at a very high level, for instance on the basis of high vs. low motivational value (e.g., Leland and Pineda, 2006, 2011).

Specifically, to investigate when and how attention is biased toward space near the hand, we used a visual target detection task in which the hand was placed near or held far from target and non-target stimuli. Targets appeared with the same frequency as non-targets (50/50) and the stimuli were counterbalanced across subjects with respect to which shapes served as targets and which served as non-targets. The matching of targets and non-targets for frequency and visual features is critical to the paradigm because it allows us to determine, without confounding factors, whether hand effects on ERP components are selective to targets or apply to non-targets as well. The P3 is thought to reflect post-categorical processing and is sensitive to motivation and task-demands. If visual stimuli appearing near the hand evoke relatively larger P3 components, we would expect larger or exclusive hand effects for target stimuli as compared with non-target stimuli. The Nd1 is an early component that is not clearly pre- or post-categorical but appears to be sensitive to cross-modal influences. Given prior findings of cross-modal effects on N1-type components, we would predict that the Nd1 would show enhancement effects of hand proximity that may or may not be selective for target stimuli. Finally, because the P1 is thought to reflect early sensory processing at pre-categorical levels, if visual stimuli appearing near the hand evoke relatively larger P1 components, we expect these hand effects to be observed for both target and non-target stimuli.

## Methods

### Participants

Nineteen healthy right-handed participants (12 male, age = 20.22, *SD* = 2.95 years) completed the experiment for partial course credit. All reported normal or corrected-to-normal vision and none reported previous head trauma. The experiment was approved by the Claremont McKenna College and Scripps College Institutional Review Boards. Two participants' data were excluded from analyses due to excessive artifact.

### Stimuli and apparatus

**Figure 1:**
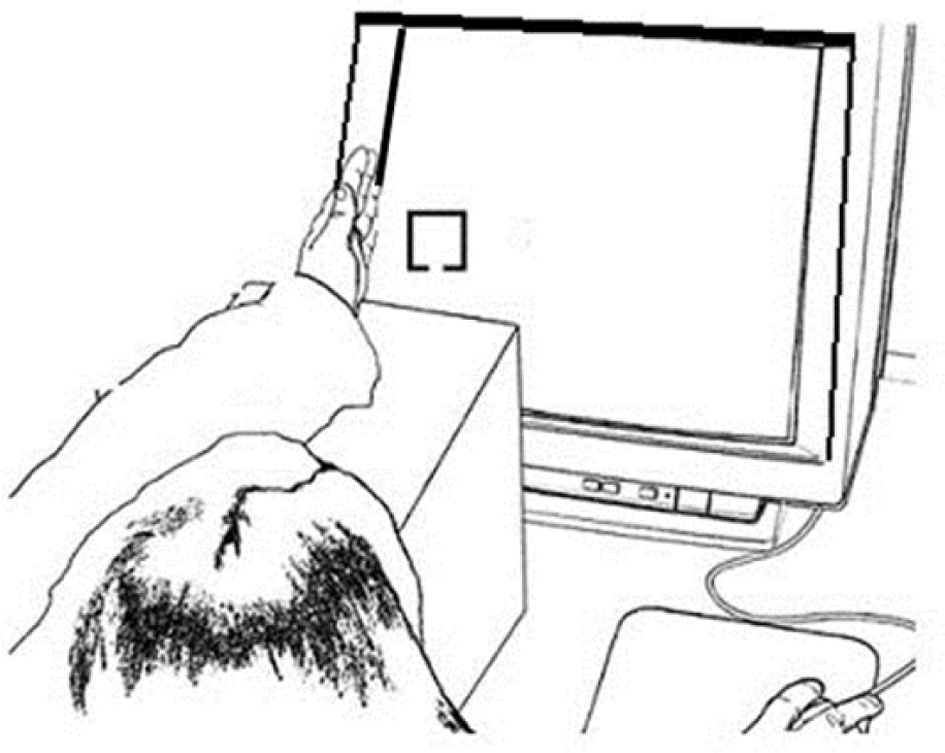
**Example experimental setup for the Hand Near stimulus Left condition**. The left hand was held on the edge of the monitor screen edge and the left fixation and stimulus appears on the left side of the screen close to the hand; the right hand responded to the target.

Stimuli were presented on a 17″ CRT monitor via a PC computer using E-Prime 1.1 software (Psychological Software Tools, Pittsburgh, PA). Responses were recorded by a PSTnet SRbox. Fixation consisted of an 8.5 × 8.5 cm dotted gray cross against a black background. Target and non-target stimuli were 2 × 2 cm yellow boxes with a 0.5 cm gap centered on either the top or the bottom border of the box against a black background. One gap location was used for targets and the other for non-targets, counterbalanced across participants. All fixation crosses and stimuli were presented at vertical center and approximately 10 cm in from the left or right side edge of the display. This allowed the left or right hand, respectively, to be placed on the plastic edge of the display monitor so that stimuli appeared near the palm, within grasping space (Figure 1). Using a Thor Laboratories Optical Power Meter (model PM100) with a S130A (400–100 nm) sensor meter held 2.54 cm in front of the monitor screen, we established that targets and non-targets registered the same power (108 mW; “mW” = milliwatts or dBm of optical power), which was higher than that for the fixation (87 mW). When the sensor was held 2.54 cm in front of the plastic edge of the monitor while fixations and stimuli alternated, we established that the sensor reading (10 mW) did not change, regardless of whether the hand was near the plastic edge of the screen or not.

### Procedure

**Figure 2:**
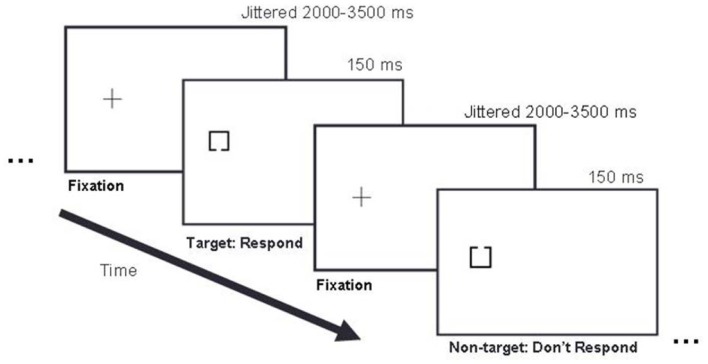
**Example trial sequence**. In this left stimulus side example, a fixation cross appeared on the left side of the display for 2000–3500 ms, followed by either a target or non-target stimulus presented for 150 ms, followed by the next fixation cross presented for 2000–3500 ms, and the next stimulus for 150 ms, and so on. Targets and non-targets appeared with equal probability (50/50) in pseudo randomized order.

Participants sat in a darkened room with their heads 50 cm from the display. Their body and shoulders were positioned square to the screen so that body midline was aligned with the center of the screen. They performed a target detection task in which 50% of trials were targets and 50% of trials were non-targets (Figure 2).

Hand position and stimulus side varied to create four conditions (Hand Near Left, Hand Near Right, Hand Far Left, Hand Far Right): Stimuli were presented either on the left or right side of the screen and the same-side hand was placed either near or far from the stimulus location (e.g., the left hand was held near or far from the left stimuli and the right hand was held near or far from the right stimuli). The index finger of the opposite-side hand was used to make button presses. For each block, participants either placed one hand on the edge of the monitor next to the fixation cross with the thumb directed up in a relaxed grasping position (Hand Near), or placed the hand in the lap (Hand Far). In Hand Near conditions, participants rested their elbows on a cushion and relaxed their arms and shoulders. To equate visual inputs for the two conditions, participants performed the task in a fully darkened room and, in the Hand Near conditions, placed their hands on the plastic edge of the monitor. Although previous behavioral and EEG studies have documented bias effects of hand proximity on performance when the hand is visible as well as when the hand is not visible (Reed et al., 2006; Garza et al., 2009), the experiment was conducted in a dark room so that participants could not see their hands and the light from the stimuli displayed on the monitor did not illuminate or reflect off the hand and arm.

Trials began with the appearance of a lateralized fixation cross. After a variable SOA of 2000–3500 ms, a stimulus appeared for 150 ms at the center of the cross. Participants pressed a response key with the index finger of the opposite hand if a target appeared. Following the response, or after 2000 ms post-stimulus onset, the fixation re-appeared for the next trial. Each type of block was presented four times, for a total of 16 blocks. Each block included 25 target trials and 25 non-target trials. Block order and trial order were pseudo-randomized so that two blocks of the same type could not follow each other and no more than four trials in a row occurred with the same stimulus type (target or non-target). Participants received feedback on performance accuracy at the end of each block and were given brief breaks between blocks.

### ERP recording

EEG was acquired using a high-impedance EGI 64-channel Hydrocel Geodesic EEG System (GES) 200 (Electrical Geodesic Inc., Eugene, OR, USA). The EOG was recorded from electrodes located above and below each eye. The EEG sampling frequency was 250 Hz with a hardware band-pass filter from 0.1 to 100 Hz. Impedances were kept below 80 Ω.

EEG and EOG data were processed off-line using NetStation 4.4.2 (Electrical Geodesic Inc., Eugene, OR, USA). Data were filtered with a 35 Hz low-pass filter. Continuous data were segmented from −100 ms pre-stimulus onset to 800 ms post stimulus onset for eight conditions: 2 (Hand Near, Hand Far) × 2 (left, right) × 2 (target, non-target). Only data from correct trials were analyzed. Data were visually inspected for blinks and eye-movements after an automatic artifact rejection criterion of ±140 μV was applied from –100 pre-stimulus onset to 800 ms post-stimulus onset. NetStation's Ocular Artifact Removal tool (Gratton et al., 1983; Gehring and Foote, 1996) was used with a blink slope threshold of 13 μV/ms to correct and remove ocular artifact. Surviving trials were averaged by condition relative to a baseline of −100 to 0 ms. Data were re-referenced using an average reference.

## Results

### Response time analyses

**Figure 3:**
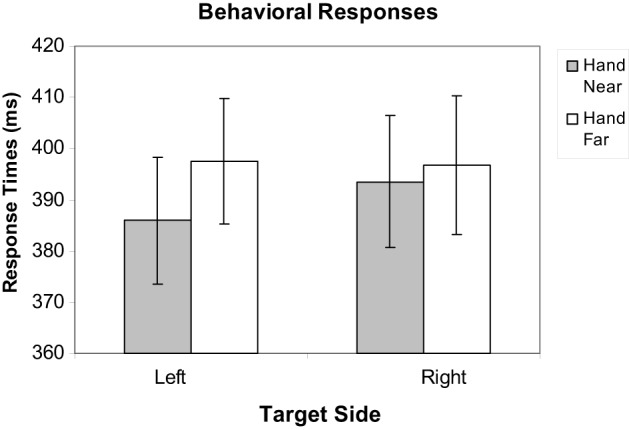
**Mean response time data for Hand Near and Hand Far conditions for targets appearing on left and right sides of the display**. Error bars represent standard error.

Mean response times (RTs) for correct target trials were calculated, excluding misses and trials with RTs outside of a window of 200–650 ms to factor out preemptive responses or inattention errors, as in Reed et al. (2006, 2010) and Garza et al. (2013); fewer than 1% of trials were excluded. To evaluate the effect of hand position on target RTs, a repeated-measures hand position (2: Hand Near, Hand Far) × stimulus side (2: left, right) analysis of variance (ANOVA) was conducted. Participants responded faster for targets near the hand than targets far from the hand [*F*_(1, 16)_ = 6.44, *p* = 0.022, η^2^_*p*_ = 0.29; Figure 3]. No main effects were found for hand side or the hand position by hand-side interaction (*p*'s > 0.31).

### ERP analyses

**Figure 4:**
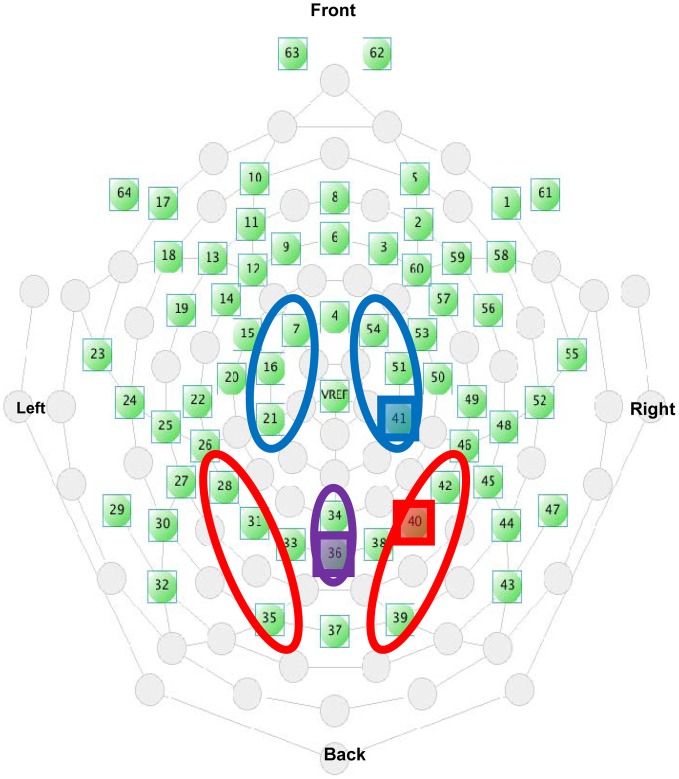
**Electrode selection from the EGI 64-channel Hydrocel net**. The highlighted electrodes indicate the placement of the representative electrodes used in Figures 5B, 6B, and 9.

Electrode sites are identified using the international 10–10 system (Figure 4). Electrode clusters and latency windows were chosen based on those reported in visuo-tactile multisensory integration studies (Kennett et al., 2001; Simon-Dack et al., 2009), visual attention studies (Eimer, 1994), and from an examination of where deflections were most prominent in the grand average waveforms sites from the current data set: P1 (80–110 ms) for lateral parietal-occipital sites (O1/P1/P3; O2/P2/P4), central Nd1 (120–190 ms) for midline parietal-occipital sites (Pz/POz), P3 (350–450 ms,) and a late positivity referred to as the P3Termination or P3T (450–650 ms) for lateral central-parietal sites (FC1/C1/CP1; FC2/C2/CP2). Mean amplitude values were calculated within specified time windows.

Within-subjects ANOVAs were conducted for each ERP component and for target and non-target stimuli, using the following factors: hand position (2: Hand Near, Hand Far), stimulus side (2: Left, Right), and, for the lateralized P1, P3 and P3T components, cluster location/hemisphere [2: Left Hemisphere (LH), Right Hemisphere (RH)].

#### P1 analyses

**Figure 5:**
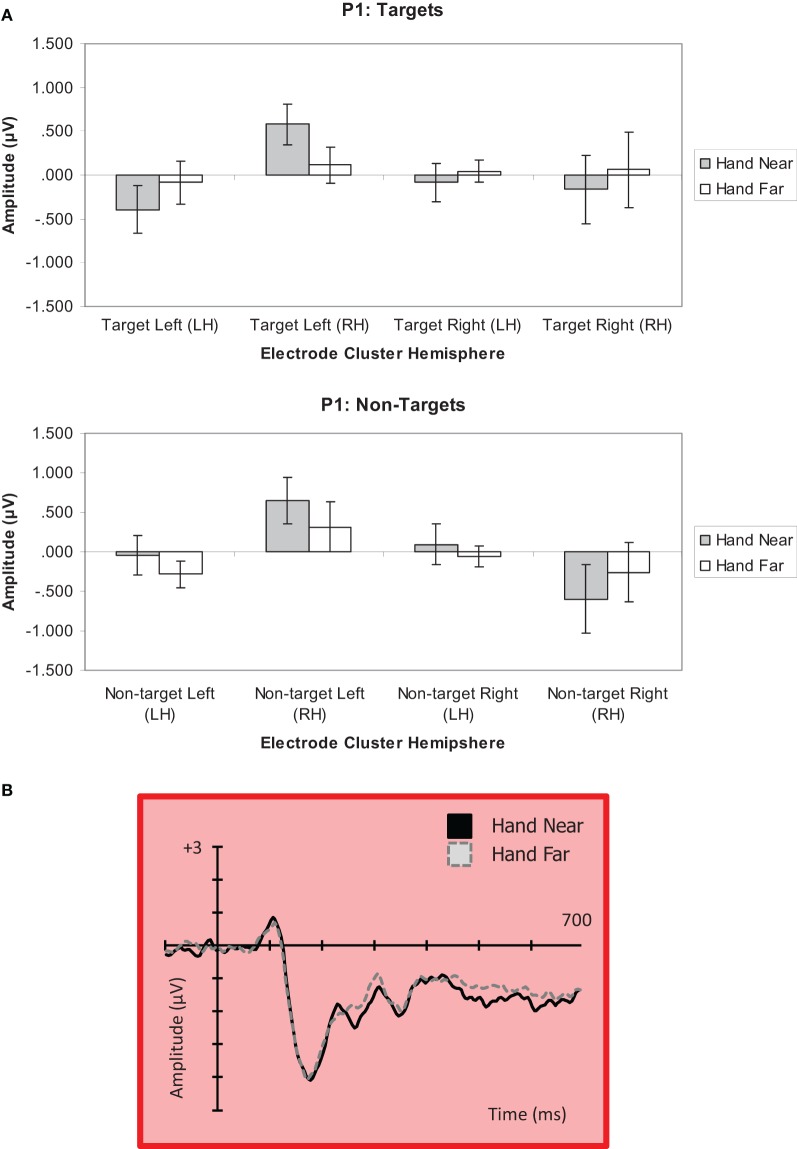
**(A) P1 (80–110 ms) mean amplitudes (μV) comparing Near and Far conditions for left- and right-side targets and non-target stimuli in left (LH) and right hemisphere (RH) electrode clusters**. Error bars represent standard error. **(B)** Grand average waveform for the P1 at representative electrode site P2 in the contralateral right hemipshere. Voltage is plotted as a function of time, 100 ms pre-stimulus onset to 700 ms post-stimulus onset. The left Near condition produced a significant effect in the right hemisphere.

***Targets.*** For targets, a significant hand position × stimulus side × cluster interaction revealed generally larger P1 amplitudes in electrode clusters contralateral to target side, and greater P1 deflections in RH electrode clusters for left targets in the Hand Near condition [*F*_(1, 16)_ = 5.13, *p* = 0.04, η^2^_*p*_ = 0.24; Figure 5]. *Post-hoc* comparisons confirmed a significant hand position difference for left-side targets in the contralateral RH cluster [*t*_(16)_ = 2.87, *p* = 0.01], but not for other hand position comparisons (*p's > 0.13).* No other main effects or interactions were found (all *p*'s > 0.09).

***Non-targets.*** A significant hand position × stimulus side × cluster interaction was found for non-targets [*F*_(1, 16)_ = 4.44, *p* = 0.05, η^2^_*p*_ = 0.22; Figure 5]. A stimulus side main effect indicated larger amplitudes for left-side relative to right-side non-targets [*F*_(1, 16)_ = 5.23, *p* = 0.04, η^2^_*p*_ = 0.25]. Also, a stimulus side × cluster interaction showed larger P1 deflections for clusters contralateral to stimulus side [*F*_(1, 16)_ = 10.34, *p* = 0.005, η^2^_*p*_ = 0.39]. *Post-hoc* comparisons indicated a trend for larger P1 amplitude in the Hand Near than Hand Far condition for left-side targets in the contralateral RH cluster [*t*_(16)_ = 1.73, *p* = 0.10]. A larger negative deflection was found in the Hand Far than Hand Near condition for right-side non-targets in the ipsilateral RH cluster [*t*_(16)_ = −2.70, *p* = 0.02], but it does not fit the pattern established by our other results, which overall show a larger effect for stimuli near the hand and/or over the hemisphere contralateral to the stimulus (for lateralized potentials). No other Hand Near/Far comparisons were significant (all *p*'s > 0.29). No other main effects or interactions were found (all *p*'s > 0.11).

#### Nd1 analyses

**Figure 6:**
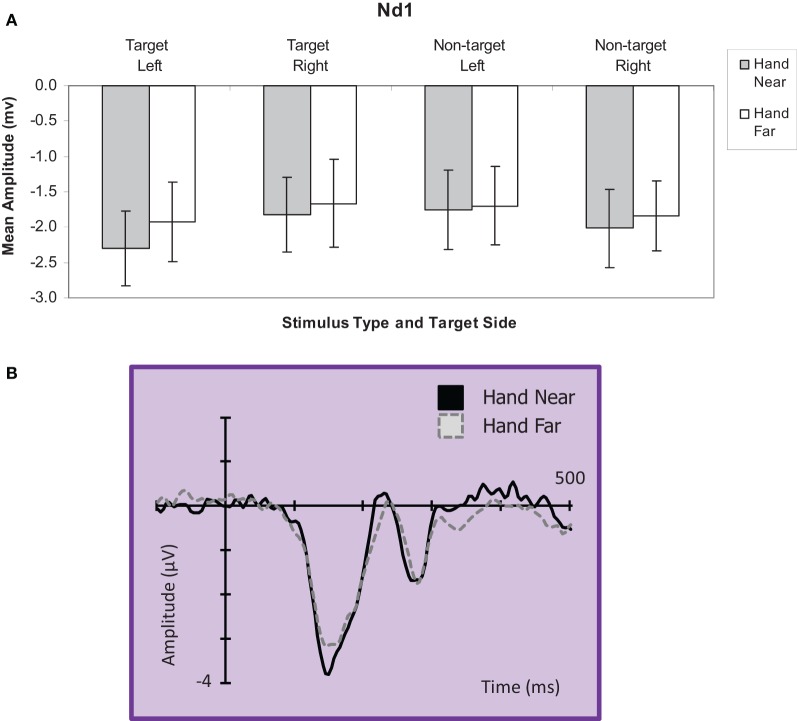
**(A) Nd1 (120–190 ms) mean amplitudes (μV) comparing Hand Near and Hand Far conditions for left and right-side target and non-target stimuli for the central electrode cluster**. Error bars represent standard error. **(B)** Grand average waveform for the Nd1 at representative electrode site POz. Voltage is plotted as a function of time, 100 ms pre-stimulus onset to 700 ms post-stimulus onset. Compared to Hand Far conditions, Hand Near conditions produced a larger Nd1.

***Targets.*** A hand position × stimulus side ANOVA for the Nd1 site (Kennett et al., 2001) revealed a significant hand position effect for target stimuli [*F*_(1, 16)_ = 4.90, *p* = 0.04, η^2^_*p*_ = 0.23; Figure 6], showing greater deflections for Hand Near compared to Hand Far conditions. A stimulus side effect indicated greater Nd1 amplitudes for targets appearing on the left than targets on the right [*F*_(1, 16)_ = 4.49, *p* = 0.05, η^2^_*p*_ = 0.22]. There was no interaction [*F*_(1, 16)_ < 1, *p* = 0.49, η^2^_*p*_ = 0.03].

***Non-targets.*** For non-target stimuli, no main effects or interactions reached significance (all *p*'s > 0.28; Figure 6).

#### P3 analyses

**Figure 7:**
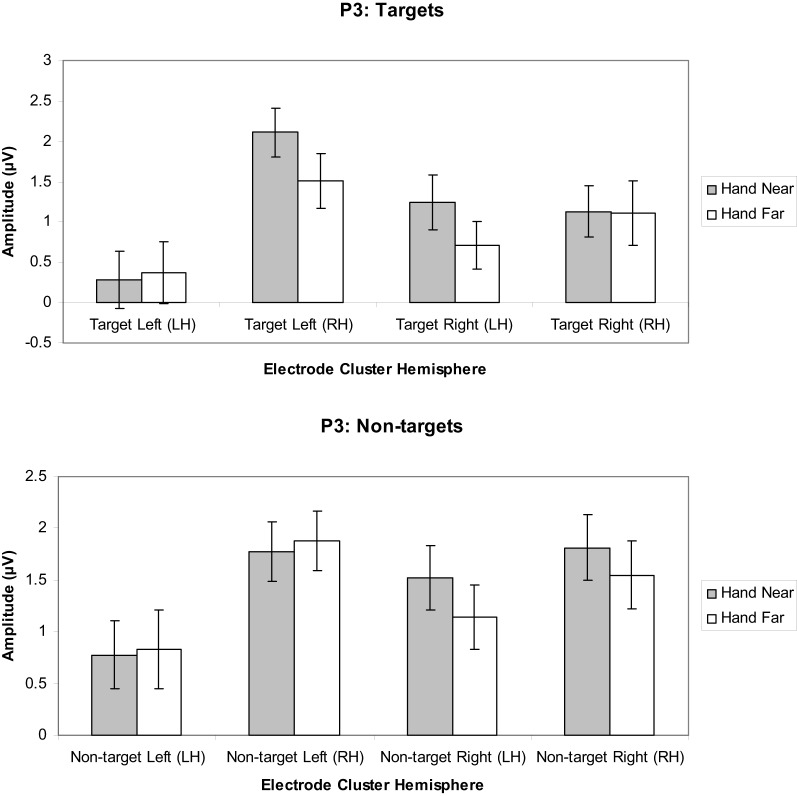
**P3 (350–450 ms) mean amplitudes (μV) comparing Hand Near and Hand Far conditions for left- and right-sided target and non-target stimuli in left (LH) and right hemisphere (RH) electrode clusters**. Error bars represent standard error. For both left- and right-side targets, P3 amplitudes for the Near condition were larger relative to the Far condition in the contralateral hemisphere.

***Targets.*** A significant hand position × stimulus side × cluster interaction indicated larger positivities in the RH electrode clusters overall, but also larger contralateral positivities for the Hand Near condition, especially in the LH electrode clusters [*F*_(1, 16)_ = 11.60, *p* = 0.004, η^2^_*p*_ = 0.42; Figures 7, 9]. *Post-hoc t*-tests showed significant hand position differences in the contralateral hemisphere [right target/LH *t*_(16)_ = 2.36, *p* = 0.03; left target/RH *t*_(16)_ = 4.55, *p* < 0.0001], but not the ipsilateral hemisphere [right target/RH *t*_(16)_ = 0.66, *p* = 0.95; left target/LH *t*_(16)_ = −0.36, *p* = 0.72]. The trend for hand position [*F*_(1, 16)_ = 3.67, *p* = 0.07, η^2^_*p*_ = 0.19] revealed a tendency for larger P3 amplitudes for Hand Near than Hand Far positions. There was a cluster side/hemisphere effect, suggesting greater P3 amplitudes in the RH than LH electrode clusters [*F*_(1, 16)_ = 4.70, *p* = 0.05, η^2^_*p*_ = 0.23]. The stimulus side × hemisphere interaction [*F*_(1, 16)_ = 12.97, *p* = 0.002, η^2^_*p*_ = 0.45] was mediated by the three-way interaction reported above. No effects were found for stimulus side, hand condition × stimulus side interaction, or the hand condition × hemisphere interaction (all *p*'s > 0.63).

***Non-targets.*** A marginal effect for cluster side/hemisphere [*F*_(1, 16)_ = 3.39, *p* = 0.08, η^2^_*p*_ = 0.18] suggested greater amplitudes for RH over LH clusters (Figure 7). No other main effects were found [hand position: *F*_(1, 16)_ = 1.25, *p* = 0.28, η^2^_*p*_ = 0.07; stimulus side: *F*_(1, 16)_ = 2.58, *p* = 0.13, η^2^_*p*_ = 0.14]. A stimulus side × cluster interaction indicated relatively larger amplitudes in the contralateral hemispheres [*F*_(1, 16)_ = 5.80, *p* = 0.03, η^2^_*p*_ = 0.26], but none of the other interactions reached significance (all *p*'s > 0.13).

#### P3T analyses

**Figure 8:**
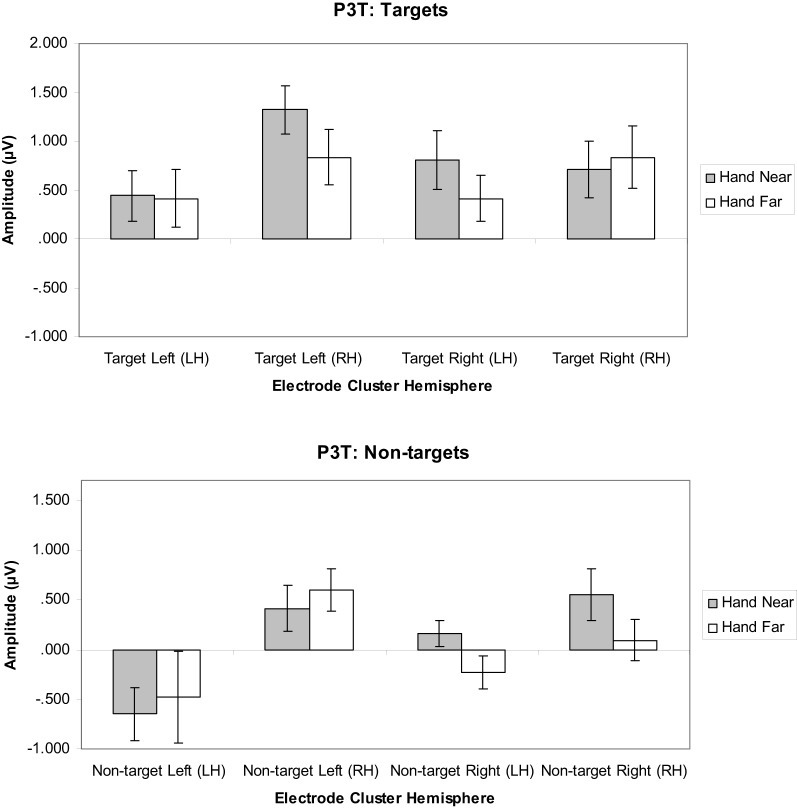
**P3T (450–600 ms) mean amplitudes (μV) comparing Hand Near and Hand Far conditions for left- and right-sided target and non-target stimuli in left (LH) and right hemisphere (RH) electrode clusters**. Error bars represent standard error. For both left- and right-side targets, Near condition P3T amplitudes continued to be larger relative to the Far condition in the contralateral hemisphere.

**Figure 9:**
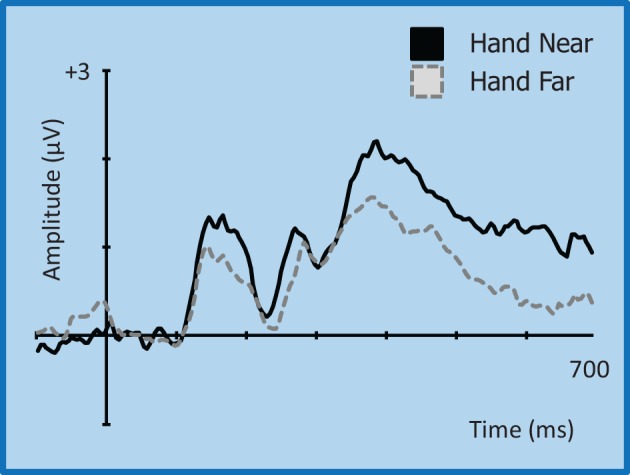
**Grand average waveform for the P3 (350–450 ms)/P3T (450–600 ms) for left-side targets at representative electrode site CP2 in the contralateral right hemisphere**. Voltage is plotted as a function of time, 100 ms pre-stimulus onset to 700 ms post-stimulus onset. Compared to Hand Far conditions, Hand Near conditions show an amplification of the P3 component and a greater sustained positivity for the P3T, especially in the contralateral hemisphere.

***Targets.*** A significant hand position × stimulus side × cluster interaction indicated that the hand had continued processing influences [*F*_(1, 16)_ = 11.19, *p* = 0.004, η^2^_*p*_ = 0.41; Figures 8, 9]. Similar to the P3, the prolonged positivity showed greater P3T amplitudes the RH electrode clusters overall and for Hand Near conditions in the contralateral hemisphere relative to the Hand Far conditions. *Post-hoc t*-tests showed significant hand position differences in the contralateral hemisphere [right target/LH *t*_(16)_ = 2.24, *p* = 0.04; left target/RH *t*_(16)_ = 3.43, *p* = 0.003], but not the ipsilateral hemisphere [right target/RH *t*_(16)_ = −0.95, *p* = 0.36; left target/LH *t*_(16)_ = 0.13, *p* = 0.90]. There were no other significant main or interaction effects (all *p*'s > 0.10).

***Non-targets.*** A cluster side/hemisphere effect showed larger amplitudes for RH than LH clusters [*F*_(1, 16)_ = 6.82, *p* = 0.02, η^2^_*p*_ = 0.30; Figure 8]. No other main effects and interactions were significant (all *p*'s > 0.08).

## Discussion

The hand may capture attention because of its relevance to future actions. For many future actions it is important to know what stimuli to act upon and which ones to ignore. In this study we examined behavioral and electrophysiological responses to characterize differential hand position influences on the neural processing of visual stimuli when they were and were not relevant to the task. We used a target detection paradigm in which non-target stimuli had the same probability and (through counter-balancing) visual features as target stimuli. The hand was placed either nearby, with the palm facing stimuli, or far away in the lap. Consistent with previous behavioral studies, RTs were facilitated for targets appearing near the hand compared to far from the hand. Nonetheless, the examination of hand position effects on the P1, Nd1, P3, and P3T ERP components demonstrated not only when but also the circumstances under which hand-related attentional biases occur. Distinguishing the neural signatures between target and non-target stimuli revealed the extent to which the hand's influence on processing was selective for goal/task-relevant stimuli as opposed to nonselective and applicable to all stimuli.

Our major ERP finding was that Nd1 and P3 amplitudes were modulated by the hand for target stimuli only; targets near the hand evoked larger potentials than targets far from the hand, but there was no difference for non-targets. The hand effect persisted late into the ERP (P3T: 450–650 ms). We also found evidence of a hand effect on the target-evoked P1 over the right hemisphere for targets presented on the left. There was a trend toward a similar effect for non-targets, which would be consistent with the P1 reflecting early visual processing before stimuli are discriminated as targets vs. non-targets. ERP effects in general showed a pattern of right hemisphere dominance. Collectively, these findings suggest that the hand enhances visuospatial processing over a wide temporal window, starting at the level of sensory processing as reflected by the P1 component and continuing with higher levels of cognitive processing such as stimulus discrimination (Nd1) and evaluation (P3). This effect appears selective for goal/task-related stimuli once stimuli have been categorized as such, but it may apply non-selectively at earlier stages of processing.

An overall examination of the ERPs elicited in response to both target and non-target stimuli revealed enhanced amplitudes for stimuli presented near the hand, especially for electrode clusters contralateral to the hand and stimulus side. Generally, right hemisphere electrode clusters were more sensitive to hand position effects. This right hemispheric dominance for the earlier components may indicate a spatial processing advantage for the right hemisphere (Picton, 1992) and has been documented previously for spatial processing, as well as for the general distribution of attention (Weintraub and Mesulam, 1987).

The first hand-related effects were observed for the P1 (80–110 ms) component. Specifically, the P1 in the right hemisphere electrode cluster was amplified for left-side targets near the hand; there was little response in the left hemisphere electrode cluster for right-side stimuli. The P1 is often considered an index of sensory processing and encoding or sensory gain (Naatanen and Picton, 1987; Naatanen, 1992). It has also been interpreted as a reflection of focused attention (Hillyard and Anllo-Vento, 1998; Martinez et al., 1999). There was also a trend for a P1 effect for non-targets that matched the one found for targets (larger for stimuli near the hand, shown on the left side, contralateral and thus over the right hemisphere). Overall, the influence of hand position on P1 amplitudes for both targets and non-targets on the P1 could suggest that the hand has an early, pre-categorical effect on sensory gain and/or attention. A possible mechanism for this effect may be contributions from bimodal neuron populations that respond to visual stimuli near the hand (e.g., Graziano and Cooke, 2006). In non-human primates, these visuo-tactile bimodal neurons have hand-centered receptive fields and respond both to tactile stimuli and visual stimuli near the hand (Graziano, 1999). Bimodal neurons in conjunction with visual neurons could facilitate visual processing by encoding the same spatial location, but would not differentiate targets and non-targets.

Although bimodal neurons have been postulated to explain behavioral hand effects (e.g., Reed et al., 2006), our finding of a contralateral P1 effect for left-side targets (and possibly non-targets) but no left hemisphere effects suggests that potential bimodal neuron effects are a weak effect at best in our experiment. Experiments designed to emphasize early visual components may produce stronger evidence for this mechanism. However, our results point to other mechanisms contributing to hand-related effects. For instance, stronger amplitudes overall in the right hemisphere support a right-hemisphere dominance for spatial processing (e.g., Hugdahl, 2013; Reed et al., 2009). In addition, hand-related effects appear to be more robust later in processing. A recent EEG study by Qian et al. (2012) that examined hand position effects on the visual evoked potential (VEP) also did not find hand-related effects on the P1, but instead found them slightly later with the P2. When both hands were up to either side of a screen (as opposed to down on the desk), the P2 was attenuated for stimuli but only in the regions of space where targets could appear. Similarly, the later components we examined (Nd1, P3) showed hand effects that were restricted to task-relevant conditions, which in our case was based on stimulus features distinguishing targets and non-targets.

We observed hand-related amplification of the central Nd1 component (120–190 ms) for both left- and right-side stimulus conditions. The Nd1 has been interpreted as an index of attentional selection and multisensory integration (Kennett et al., 2001). Specifically, increased negativities were observed for targets appearing near the hand. This amplification suggests that tactile and proprioceptive inputs regarding hand location may be integrated with inputs from the visual stimulus. This slightly later component has been associated with multisensory integration in which physical tactile stimulation occurred with congruent visual stimulation or the viewing of the limb (Spence et al., 1998; Kennett et al., 2001; Taylor-Clarke et al., 2002). Simon-Dack et al. (2009) proposed that hand-related N1 effects could reflect the operation of visuo-tactile bimodal neurons as a mechanism to help integrate multimodal sensory information in peripersonal space (Graziano and Cooke, 2006). However, our comparison of both targets and non-targets suggests a different mechanism. Our results showed hand position effects for the Nd1 for targets only, indicating that stimulus classification had already occurred. The implicit relevance of hand location relative to visual targets may bias the system toward visuo-tactile integration in a top-down fashion. In sum, the Nd1 findings indicate that by the time stimuli are discriminated, the hand effect becomes selective for attended or action-relevant stimuli (targets).

Hand position not only affected early ERP components, but also the later P3 (350–450 ms) and possibly P3T (450–650 ms) latency ranges. The P3 is typically elicited in detection tasks for which targets are presented infrequently among frequent non-target stimuli (Polich, 2007). In our study, targets and non-targets were equally frequent. Although the P3s did not appear to be as large as for paradigms with rare targets, we found that hand position modulated P3 deflections for targets but not non-targets. This indicates that hand position has a more abstract, post-categorical effect on later visual processing. This is consistent with the observation that selected stimuli near the hand receive improved processing even when hand-proximity is imagined (Davoli and Abrams, 2009). Here we observed significantly larger P3 deflections when left-side or right-side targets were presented near the hand. The hand effect was strongest for electrode clusters contralateral to the target side. The P3 is associated with short-term memory maintenance and updating of target classification information (Picton, 1992) as well as attention and goal-related processing (Polich, 2007). Thus, target stimuli presented near the hand may enjoy attention, memory, and other cognitive benefits by a mechanism similar to that for stimuli that are infrequent, task-relevant, and/or motivationally salient. That this enhanced positivity appears to persist late into the ERP (P3T, 450–650 ms) may confer further advantages supporting effective action toward objects near the hand.

Collectively, our ERP findings suggest that the hand biases processing selectively for goal/task-relevant stimuli at later stages of processing. A bias is also evident at an early sensory/perceptual stage but at that point it may be non-selective (applying to target and non-target stimuli), which is to be expected if stimuli are not discriminated on the basis of such categories until later in processing. We interpret this as evidence of both non-selective pre-categorical effects and selective post-categorical effects of hand position. Although this may correspond to bottom-up and top-down influences, respectively, it is important to note that the block design of our study promotes an attentional set. That is, in the Hand Near condition the hand is up for the entire block and all stimuli appear in grasping space, providing a top-down influence of endogenously-driven attention throughout. Studies in which participants are told to attend to one region of space and not another (e.g., Chun and Wolfe, 2001) commonly show P1 (and N1) amplitude enhancements for attended space; these are effects at early stages of the visual response but reflect top-down attention.

A theory of embodied spatial attention implies that our bodies and our experience using them influence how attention is distributed in space and, as a result, how stimuli are processed. Our findings suggest such an influence for the hand both behaviorally and electro physiologically: stimuli appearing near the hand elicit faster response times and enhanced attention-related ERP components. This latter effect is evident at both early and late stages of processing, before and after stimulus categorization. Early detection for stimuli in general and prolonged facilitation of processing specifically for goal/task-relevant stimuli provide an effective combination allowing for more adaptive action toward objects in the environment that are most important and accessible.

### Conflict of interest statement

The authors declare that the research was conducted in the absence of any commercial or financial relationships that could be construed as a potential conflict of interest.
